# PTCH1, a receptor of Hedgehog signaling pathway, is correlated with metastatic potential of colorectal cancer

**DOI:** 10.3109/03009731003668316

**Published:** 2010-07-19

**Authors:** Sihong You, Jiannong Zhou, Senqing Chen, Ping Zhou, Jinghuan Lv, Xiao Han, Yujie Sun

**Affiliations:** ^1^Key Laboratory of Human Functional Genomics of Jiangsu Province, Nanjing Medical University, NanjingPeople's Republic of China; ^2^Jiangsu Institute of Cancer Research, NanjingPeople's Republic of China; ^3^Cancer Center, Nanjing Medical University, NanjingPeople's Republic of China

**Keywords:** Colorectal cancer, hedgehog, metastasis, PTCH1

## Abstract

**Background:**

Approximately 90% of colorectal cancer (CRC) deaths arise from the metastatic dissemination of primary tumors. It is difficult to predict metastasis of colorectal cancer, especially for patients with the same pathological subtype and differentiation.

**Aims:**

To identify biomarkers for predicting CRC metastasis.

**Patients and methods:**

We collected 19 primary tumors of CRC with identical pathological subtype, differentiation, and comparable Dukes' stages from patients with matched age and gender but completely different prognosis. Patients were divided into one high-risk and one low-risk group for metastasis. The expression levels of SHH, PTCH1, and sFRP1, which are components of the Hedgehog signaling pathway, were determined by real-time reverse transcription polymerase chain reaction (RT-PCR). To investigate further the correlation between expression level of PTCH1 and metastatic potential of CRC cells, we compared the mRNA and protein levels of the PTCH1 gene in LoVo cells with high metastatic potential and in HT-29, SW480, and SW620 cells with low metastatic potential by RT-PCR and flow cytometry.

**Results:**

We found that tumor tissues in the high-risk group for metastasis expressed lower levels of PTCH1 mRNA than did those in the low-risk group. Similarly, mRNA and protein levels of PTCH1 were inversely correlated with the metastatic potential of CRC cell lines. Expression levels of SHH and sFRP1 genes did not differ between the two groups.

**Conclusion:**

Our data suggest that PTCH1 might be a potential biomarker that could discriminate CRC with high from that with low metastatic risk.

## Introduction

Colorectal cancer (CRC) is the second most prevalent cancer in developed countries and the third most prevalent cancer in developing nations (1,2). During the past few decades, the morbidity and mortality of CRC have increased rapidly in China (3). Although great progress has been made in the diagnosis and treatment, such as in colonoscopy (4), chemotherapy (5), and molecular targeted drugs (6), the 5-year mortality for people diagnosed with CRC remains approximately 40% (7). Since approximately 90% of colorectal cancer deaths arise from the metastatic dissemination of primary tumors (8,9), it is important to find biomarkers which can predict the risk of metastasis of colorectal cancer. Although a number of molecular markers have been found to be possible prognostic factors, more ‘predictors’ need to be determined which can identify patients with high risk for metastasis (10).

Wnt and Hedgehog signaling pathways are both highly implicated in colonic epithelial homeostasis. Imbalance between the two pathways is correlated to gastrointestinal cancers (11). Hedgehog family ligands include Sonic hedgehog (SHH), Indian hedgehog (IHH), and Desert hedgehog (DHH) (12,13). In the absence of Hedgehog ligand, Patched family receptors (PTCH1 and PTCH2) inhibit another membrane receptor Smoothened (SMO), which leads to the formation of cytoplasmic glioma-associated oncogene (GLI) degradation complex. Binding of Hedgehog to PTCH1 or PTCH2 releases the SMO signal transducer from Patched-dependent suppression, which promotes the nuclear accumulation of full-length GLI and leads to GLI-dependent transcriptional activation of target genes, such as GLI1, PTCH1, CCND2, and FOXL1, etc. (13–17). It has been reported that SHH can promote motility and invasiveness of gastric cancer cells, suggesting the involvement of SHH signaling in the metastasis of gastric cancer (18).

In order to investigate the role of Hedgehog signaling pathway in the metastasis of colorectal cancer and find biomarkers for predicting metastasis of CRC, we analyzed the expression levels of several components of Hedgehog signaling pathway, including SHH (a Hedgehog family ligand), PTCH1 (a receptor of Hedgehog signaling pathway), and sFRP1 (a target gene of Hedgehog signaling pathway), in the primary tumors of colorectal cancers from 8 patients with low risk (no recurrence or metastasis was observed 5 years after the operation) and 11 patients with high risk for metastasis (metastasis occurred within 2 years after the operation) by using quantitative real-time reverse transcription polymerase chain reaction (RT-PCR) method. All the samples presented identical pathological subtype and differentiation, and the two groups possessed comparable Dukes' stage. To investigate further the relationship between the expression level of PTCH1 and the metastatic potential of CRC cells, we compared mRNA and protein levels of PTCH1 gene in CRC cell lines with different metastatic potentials by RT-PCR and flow cytometry.

## Materials and methods

### Patients and samples

A total of 19 primary CRC tumor tissues with identical pathological subtype (adenocarcinoma), same differentiation (Grade 2, moderately differentiated), and comparable Dukes' stages were collected from Jiangsu Institute of Cancer Research between June 2000 and June 2004. The patients were divided into two groups: patients diagnosed with metastasis within 2 years after the operation were defined as high-risk group for metastasis, and patients without recurrence or metastasis in 5 years after resection were defined as low-risk group for metastasis. None of the patients received chemotherapy or radiotherapy before operation. Among the patients, 9 cases were male, and 10 were female. The ages of the patients were from 30 to 73 and average 53.6 ± 12.2 years. The locations of the primary tumor were 9 at rectum, 4 at sigmoid, 2 at ascending colon, 2 at cecum, 1 at splenic flexure, and 1 at anal canal. Altogether 12 patients belonged to Dukes' B stage and 7 to Dukes' C stage. The site of metastases included lung (4 cases), liver (3 cases), lung and liver (2 cases), lung and bone (1 case), and ovary (1 case). The mean time interval between the operation of the primary tumor and the detection of metastasis was 16 months. The samples were frozen and stored in liquid nitrogen.

### Quantitative real-time RT-PCR

Three components of the Hedgehog signaling pathway, SHH, PTCH1, and SFRP1, were selected for quantitative real-time RT-PCR analysis. The reference sequences for each gene were obtained from the NCBI Entrez web site. The primer pairs (Table I) were designed using Primer Express (Applied Biosystems, CA, USA).

**Table I.** Sequences of primer pairs.

Genes		Primers (5'→3')	Size (bp)	GeneBank
SHH	F	CAATTACAACCCCGACATCATA	194	NM_000193
	R	CCTCGTAGTGCAGAGACTCCTC		
PTCH1	F	GGGTGGCACAGTCAAGAACAG	108	NM_000264
	R	TACCCCTTGAAGTGCTCGTACA		
sFRP1	F	TCTACTGGCCCGAGATGCTT	147	NM_003012
	R	TGGCCTCAGATTTCAACTCGTT		
β-Actin	F	GAAATCGTGCGTGACATTAA	179	NM_001101
	R	AAGGAAGGCTGGAAGAGTG		

Total RNA was extracted from tissue samples using TRIZOL^®^ Reagent (Invitrogen Life Technologies, CA, USA). First-strand cDNA was synthesized using ReverTra Ace (TOYOBO, Osaka, Japan) with 1 μg of total RNA in 20 μL reaction volume, containing 1 × Reverse Transcription Buffer, 1 mM dNTPs each, 0.5 mM Oligo(dT)_20_, 10 U RNase inhibitor, 1 μL ReverTra Ace.

Real-time PCR was carried out on an ABI PRISM 7000 system (Applied Biosystems, CA, US) using SYBR^®^ Green Realtime PCR Master Mix (TOYOBO, Osaka, Japan). PCR products were detected with melting curve analysis. The ABI PRISM 7000 SDS software (version 1.0) was used for the analysis and interpretation of the results. The reactions included 2 μL cDNA (1:10 dilution), 4 pmol of each primer and the SYBR^®^ Green Realtime PCR Master Mix. The final reaction volume was 20 μL. PCR was performed under the following cycling conditions: an initial denaturation step at 95°C for 1 min, followed by 40 cycles of amplification (95°C for 15 s, 60°C for 15 s, and 72°C for 45 s). Every PCR reaction of each gene and each sample was done in duplicate.

Real-time PCR data were quantitated using the cycle threshold (CT) method. β-Actin mRNA was used as an internal control. The amount of target genes mRNA in tumor tissue relative to that in paired self control was calculated as follows:

(Eq.1) ΔCT(Tumor or Control)=CT(Target Gene)−CT(β-actin)

(Eq.2) ΔΔCT=ΔCT(Tumor)−ΔCT(Control)

(Eq.3) $$\text{Amount of target}=2^{-\text{ΔΔCT}}(19)$$

Greater 2^–ΔΔCT^ of a certain gene in a paired tumor and control samples represents a higher ratio of mRNA quantity in tumor over control.

### Cell culture

LoVo cell line with high metastatic potential and HT-29, SW480, and SW620 cell lines with low metastatic potential were obtained from ATCC (American Type Culture Collection, Manassas, VA, USA). HT-29, SW480, and LoVo were maintained in RPMI 1640 (Gibco, CA, USA), supplemented with 10% (v/v) new-born calf serum, and SW620 was maintained in Leibovitz's L-15 (Gibco, USA), supplemented with 10% (v/v) fetal bovine serum. All media contained streptomycin (100 U/mL) and penicillin (100 U/mL). The cells were grown at 37°C in a humidified 5% CO_2_ atmosphere.

### Wound-healing assay

Wound-healing assays were performed to evaluate the metastatic potential of CRC cells. HT-29, SW480, SW620, and LoVo cells were seeded in 12-well plates at a density of 4 × 10^5^ cells/well and cultured for 12–24 h until they reached 90% confluence before scratching across the cell monolayer with a plastic tip. Cells were then grown in serum-free medium. Wound-healing was observed at every 6 hours within the scrape line, and representative scrape lines were photographed. Quantification of the area was performed using Image-Pro Plus 4.5, and cell motility was calculated as follows:

(Eq.4) $$\frac{\left(\text{initial wound area}-\text{remaining wounded area}\right)}{\text{initial wound area}}\times \%$$

### Detection of PTCH1 mRNA by RT-PCR in cell lines

Total RNA was extracted from cells in exponential growth phase. First-strand cDNA was synthesized according to the protocol mentioned above. PCR conditions for PTCH1 gene were 94°C for 5 min; 40 cycles of 94°C for 30 s, 60°C for 30 s, and 72°C for 30 s; and a final extension at 72°C for 10 min in a volume of 50 μL, containing 1 × PCR Buffer, 2.5 μL of cDNA, 0.5 μM of each primer, 0.2 mM dNTPs each, 1.5 mM magnesium chloride, and 1.25 units of Taq DNA Polymerase (TaKaRa BioTechnology, Dalian, China). The PCR products were separated on 2% (w/v) agarose gels.

### Flow cytometry analysis

Rabbit polyclonal to Patched/PTCH primary antibody (Abcam, Cambridge, UK) was used to label PTCH1 protein on CRC cells. Adherent cell monolayers were detached by treatment with 0.25% trypsin and 0.02% EDTA. After fixation in 1% paraformaldehyde for 20 minutes, cells were stained with primary antibody at 1:100 dilutions overnight, at 4°C. After washing with phosphate-buffered saline (PBS), the cells were incubated with fluorescein isothiocyanate (FITC) conjugated goat anti-rabbit IgG antibodies (1:100) for 1 h at room temperature in the dark. Flow cytometry analysis was then performed on a FACSCalibur (BD Biosciences, San Jose, CA, USA), and 10,000 events were collected and analyzed with CellQuest software (BD Biosciences). Base-line staining was obtained by adding PBS to the cells instead of primary antibody. Additional negative controls included irrelevant antibodies that confirmed the specificity of the staining.

### Statistical analysis

Statistical analyses were carried out using the SPSS program (SPSS, version 11.0, Chicago, IL, USA) and Microsoft Excel 2003 (Microsoft Corporation, Seattle, USA). Comparison of data between high-risk group and low-risk group for metastasis utilized Mann-Whitney *U* test. For comparison of wound-healing assay and flow cytometry data between CRC cells, independent sample *t* tests were carried out.

## Results

### Expression levels of PTCH1 in colorectal cancer samples

**Figure 1.**
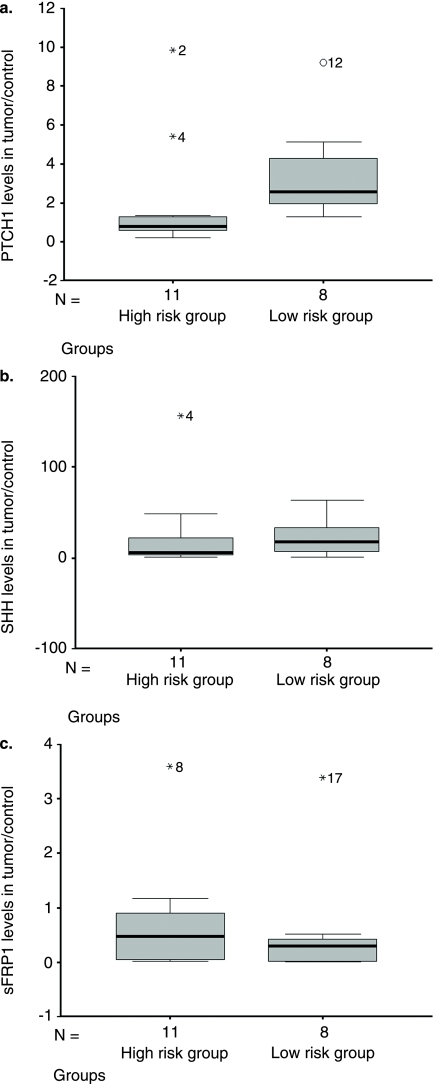
Expression level of a target gene was determined by real-time PCR. Calculation of statistical difference between high-risk group and low-risk group utilized Mann-Whitney *U* test.

To identify biomarkers that could discriminate patients with high risk for metastasis from those with low risk, we collected 19 primary tumor samples with identical pathological subtype, same differentiation, and comparable Dukes' stages from CRC patients. The patients were divided into one high-risk and one low-risk group for metastasis. There was no difference in gender, age, and Dukes' stage between the two groups. Expression levels of SHH, PTCH1, and sFRP1 mRNA in cancer samples were analyzed with real-time PCR. We found that the high-risk group had lower levels (*P* = 0.002) of PTCH1 mRNA than did the low-risk group (Figure 1A). However, expression levels of the SHH and sFRP1 genes did not differ between the two groups (Figures 1B–C).

### Expression level of PTCH1 mRNA in cells with high and low metastatic potential

**Figure 2.**
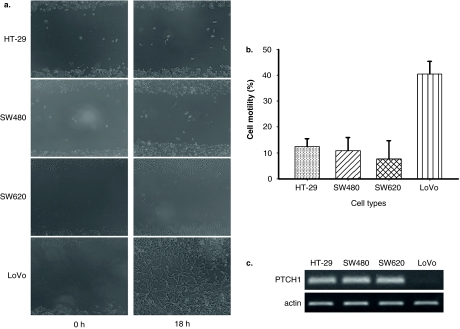
A: HT-29, SW480, SW620, and LoVo cells were seeded in 12-well plates and cultured for 12–24 h until they reached 90% confluence before scratching across the cell monolayer with a plastic tip. Cells were then grown in serum-free medium. Wound-healing was observed at every 6 hours within the scrape line, and representative scrape lines were photographed. B: Quantitative analysis of three independent cell motility experiments with LoVo, HT-29, SW480, and SW620 cells. C: RT-PCR experiments to demonstrate expression levels of PTCH1 mRNA in the same cell lines.

To investigate the correlation of expression level of PTCH1 and metastatic potential of CRC cells further, we examined the expression level of PTCH1 mRNA by RT-PCR in LoVo, HT-29, SW480, and SW620 cells. The metastatic potential of these cells was determined by wound-healing experiments. After 18 hours of incubation, LoVo cells filled almost 40% of the scratched area compared with approximately 10% by HT-29, SW480, and SW620 cells (Figures 2A–B). RT-PCR revealed that the expression level of PTCH1 mRNA was lower in high metastatic LoVo cells than that in low metastatic HT-29, SW480, and SW620 cells (Figure 2C).

### Expression level of PTCH1 protein in cells with high and low metastatic potential

**Figure 3.**
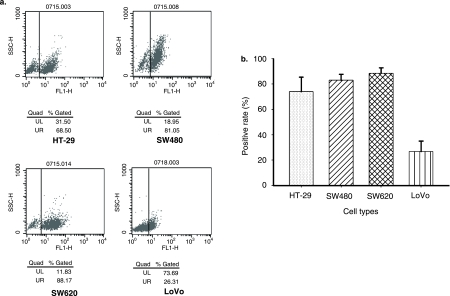
Flow cytometry analysis of different cell lines. Rabbit polyclonal to Patched/PTCH primary antibody was used to label PTCH1 protein on CRC cells. FITC-conjugated goat anti-rabbit IgG antibody was used as secondary antibody. A: Typical flow cytometric dot plots. B: Histograms show results of three independent experiments, and bars indicate standard deviations (independent sample *t* test).

The correlation between expression level of PTCH1 protein and metastatic potential of CRC cells was further investigated in HT-29, SW480, SW620, and LoVo cells by flow cytometry analysis using an anti-PTCH1 antibody. As expected, the positive rates for PTCH1 were significantly higher in HT-29, SW480, and SW620 cells with low metastatic potential than that in LoVo cells with high metastatic potential (Figure 3A, 3B).

## Discussion

Metastasis is a common event in colorectal cancer, especially metastasizing to the liver, which leads to a failure of the therapeutic regimen (8). It is still difficult to predict metastatic risk of the disease based on clinical parameters, as colorectal cancers with identical pathological subtype, differentiation, and Dukes' stage could have significant differences in metastatic potential (10). Our study revealed that PTCH1 expression was inversely correlated with metastasis of CRC, which suggested that it could be a potential biomarker for predicting the metastatic risk of colorectal cancer. The sensitivity and specificity of PTCH1 for predicting metastatic risk of colorectal cancer should be evaluated further in larger samples. Multiple center co-operation and long-term follow-up would be necessary to get enough CRC patients with identical pathological subtype, differentiation, Dukes' stage, matched age and gender, but different metastatic risks.

Hedgehog signaling pathway plays a role in initiation and progression of gastrointestinal cancers. Hedgehog signaling is frequently activated in esophageal, gastric, and pancreatic cancer (13,20,21). PTCH1, a receptor of Hedgehog signaling pathway, can suppress the pathway by inhibiting SMO. Binding of SHH, a Hedgehog family ligand, to PTCH1 leads to release of SMO signal transducer and activation of the pathway (13,14). Loss of PTCH1 is known to release the SMO signal transducer from Patched-dependent suppression and further activates Gli1 (22). It is reported that Gli1 up-regulates the expression of Snail, which is a repressor of E-cadherin gene expression (23–25). As E-cadherin has been implicated in CRC metastasis (26), our observation implied that PTCH1 might be involved in metastasis of colorectal cancer possibly through affecting the expression of E-cadherin.

SHH signaling has been implicated in the metastasis of gastric cancer and pancreatic adenocarcinomas (18,27). Inhibition of SHH signaling could reduce the tumor burden and metastasis in pancreatic adenocarcinomas (27). sFRP1, a target gene of Hedgehog signaling pathway, together with sFRP2-5, DKK1-4, and WIF1 genes, encoding secreted-type WNT inhibitors, constitutes the negative regulation system of the Wnt signaling pathway (28). However, differential expressions of SHH and sFRP1 were not observed in the high-risk group versus the low-risk group for metastasis in this study. These results suggested that the function of PTCH1 in metastasis of colorectal cancer could be independent of SHH and sFRP1. It will be interesting to investigate the mechanisms by which loss of PTCH1 expression promotes the metastatic potential of CRC cells.
